# Animal actions and their involvement in human meaning-making processes in interaction

**DOI:** 10.3389/fsoc.2026.1816762

**Published:** 2026-06-12

**Authors:** Mika Simonen

**Affiliations:** Faculty of Social Sciences, University of Helsinki, Helsinki, Finland

**Keywords:** cognition, conversation analysis, dogs, interspecies pragmatics, meaning-making, pre-sequence

## Abstract

**Introduction:**

This article examines the meaning-making processes involved in how non-human animals, particularly dogs, contribute to our understanding of the concept of “a ball-throwing exercise.” Theoretically, the article aligns with and contributes to interspecies pragmatics by introducing cognitive dimensions to the field. Interspecies pragmatics is based on conversation analytic (CA) research; this article explores how CA findings on cognition can contribute to pragmatic questions on meaning-making processes in interaction.

**Methods:**

For this investigation, data were collected using GoPro action cameras attached to harnesses on three dogs, providing footage from their first-person perspective. The data, which total nearly 2 hours, were analyzed using CA methods.

**Results:**

We focus on dog-walking sessions where the dog summons the human to pick up its ball. The human arrives and either picks up the ball or leaves it on the ground to kick it later. This sequence is a pre to the exercise that occurs when the human returns the ball to the dog: the human throws or kicks the ball for the dog to catch. We identified two sequential occurrences in pre-sequences where human and non-human animal meaning-making processes intertwine: (1) the moment after the human’s expected answer in the second position arrives, allowing the dog proceeds to the third position closing turn (dog runs) or directly to the main sequence (dog remains in place), and (2) the moment when the dog initiates a repair due to the lack of an expected answer by the human. Common to these occurrences is that they highlight the dog’s expectations regarding the human’s action in the second position of the pre-sequence.

**Discussion:**

These findings suggest that cognition is sequentially situated within the relationship between the pre-sequence and the main sequence, indicating how occurrences in the pre-sequence materialize in the main sequence. Cognition is grounded in the sequential development that facilitates the entire exercise through cooperation with the other participant.

## 1 Introduction

Throwing balls, sticks, and various small items for pet dogs to fetch is a common everyday activity for many dog owners. However, dogs can also initiate ball games with humans. This article considers dogs’ active participation in organizing these games. Recent developments in the understanding of interspecies pragmatics (Peltola and Simonen, 2024) indicate that humans and non-human animals can jointly organize the progression of social interaction. Such mutual orchestrating can occur during ball games. This study elaborates on the well-known exercise of “throwing a ball to the dog” to inform pragmatic analysis about animal involvement in this process. Our interest relates to the question of meaning: How are dogs, or non-human animals in general, part of our meaning-making processes? Pragmatic analysis can offer valuable insights into this question.

Interspecies pragmatics is an interdisciplinary approach in the Humanities and Social Sciences aimed at redefinind the hierarchical boundaries between humans and non-humans (Peltola and Simonen, 2024). It expands the view of pragmatics (Verschueren, 1999), which is based on language studies informed by conversation analysis (CA) (Sidnell and Stivers, 2013). Interspecies pragmatics thus focuses on interspecies interaction, where a human speaker and an animal speaker (Cornips, 2022) can produce turns that both participants and researchers can recognize as meaningful actions (Mondada, 2018; Mondémé, 2023). Both human and animal actions can be classified into first and second pair parts of sequences. For example, Rossano (2013) identified a first action and a responsive action, while Mondémé (2023) found two sequence types: “summons-answer” and “request-compliance.” Interspecies interaction can unfold in larger sequential structures, such as sequences of sequences (Peltola and Simonen, 2024). Researchers contributing to interspecies pragmatics can investigate the relationship between human and animal actions and the practices used to accomplish them. Studies have shown that horses use their actions to negotiate joint decisions with humans, such as in training a non-competent rider (Szczepek Reed and Lundesjö Kvart, 2025), and that interspecies partners engage in greetings (Nilsson and Norrthon, 2024). A recent study by Cornips and Wels argues that, in addition to human knowledge, interspecies meaning-making requires emotions, touching, and multimodal engagement (Cornips and Wels, 2025).

Original CA studies found that social interaction is organized in turns of talk (e.g., Sacks et al., 1974). In our interspecies case, turn-taking in ball games is organized in embodied turns, which may limit other players. Consider the following: “While holding onto the ball, dogs and people entice their partner to try get the ball by moving it toward the partner, but limiting the likelihood that the partner can actually get the ball.” (Mitchell, 2015, pp. 31–32). Turn-taking organization in conversation can be seen as the “primordial site of sociality” (Schegloff, 1987, p. 102). But how is turn-taking in ball games organized without conversation? Views emphasizing an audio-based understanding of interaction (Schegloff, 2009) lack a perspective for multimodal features of interaction (see, e.g., Heath and Luff, 2013, p. 304). As noted also by Cornips and Wels (2025), multimodal features, such as embodied actions, may still benefit from the integration of audio-based materials (Simonen, 2023). From the perspective of interspecies pragmatics, the primordial site of sociality needs reworking to capture non-human animals’ (non-verbal) participation with humans.

Interspecies pragmatics is based on a tradition that emphasizes considering evidence of psychological dimensions in the description of the researched phenomenon (Verschueren, 1999, p. 271). Prior studies on interspecies pragmatics have not investigated cognition in interspecies interactions. Hence, this study contributes to the field of interspecies pragmatics by introducing cognitive perspectives. This is achieved by drawing relevant findings from CA studies on cognition (Potter and Edwards, 2013) and comparing our findings with those. By doing so, this study brings cognitive questions and tensions into the realm of interspecies pragmatics.

Interspecies pragmatics expands human-centered views by considering non-human animals. Unique among pragmatic approaches, it benefits from the finding that interspecies participants can co-create meaning without spoken or written language (Simonen, 2023). In line with recent developments in interspecies pragmatics (Simonen, 2023; Peltola and Simonen, 2024; Nilsson and Norrthon, 2024; Szczepek Reed and Lundesjö Kvart, 2025; Peltola and Grandgeorge, 2025), this article investigates evidence of animal actions involved in human meaning-making processes, particularly through interactions between humans and dogs. We ask what kind of evidence could illustrate the involvement of animal actions in human meaning-making during interaction. Such evidence would challenge the anthropocentric assumptions of approaches that deny the possibility of animal action in human meaning-making processes.

## 2 Background

Pragmatics explores language both as a form and in a relation to behavior (Verschueren, 1999). The latter perspective concerns empirical evidence of behavior that holds meaning for the participating individuals. This empirical evidence must be based on interpretation and cognitive processing (Verschueren, 1999, p. 271). Echoing ethnomethodological studies (Garfinkel, 1967), pragmatics pertains to meanings “which are ‘worked out’ on particular occasions of use” (Cruse, 2006, p. 136). Previous studies have identified several key meanings that are “worked out” in human–dog ball play. Mitchell (2015) investigated human–dog play with a particular focus on creativity in interaction. Following Henricks (2015, p. 226) in *Play and the Human Condition*, play should be broken down into its elemental components to reveal the meanings it carries. According to Mitchell (2015):

- Participation in the activity is collaborative and supports a form of friendly competitive play, shaped by the distinct projects of the human and the dog.^1^
- The other participant’s actions function as a resource for creativity; for example, each partner could act as a “sparring partner,” with an expectation of mutual improvement.
- The ball used by the dyad exhibits a degree of unpredictability in its movement, creating opportunities for both partners to initiate novel actions.
- Interspecies ball play could also include unusual actions—such as faking a ball toss—which might stimulate the recipient’s creative responses.
- The human and dog appear as equal partners who share a common orientation toward engaging in play.

It has been noted that (neo-)pragmatic research can engage in “an ongoing engagement with the otherness,” which may offer “richer forms of collective redescription” (Baert and da Carreira Silva, 2010, p. 304). Such engagement with the otherness may refer to non-human animals. Engagement with non-human animals could offer new possibilities for understanding. The authors continue: “The question [is] how we can learn from the encounter with the unfamiliar to challenge … and think differently” (Baert and da Carreira Silva, 2010, p. 305).

One way to think differently is to consider features that were once expected to be found exclusively in humans; Willgohs et al. (2023) report that recent studies in animal cognition have found evidence of non-human animals’ competencies in cooperation, problem-solving, planning, and tasks such as counting. Their own study focused on creativity in dogs and found that dogs are fluent in “producing novel ideas” (Willgohs et al., 2023, p. 8). Still, Bräuer (2014) argued that no evidence exists showing that dogs understand human intentions, implicating lack of theory of mind in dogs (Premack and Woodruff, 1978). Merritt (2021, p. 353) observed that studies on canine cognition focusing on the mechanisms of interspecies interactions have received little academic attention; because human and dog play occurs in interaction, it seems possible to investigate “the ways in which cognition is inherently *intersubjective*” (Merritt, 2021, p. 356; emphasis in original).

Thus, what happens in human and dog play is a collaborative achievement that cannot be studied using conventional methods of cognitive science (Merritt, 2021, p. 361). Originally, Harvey Sacks argued that CA should focus on how cognition is relevant in interaction and not push efforts to investigate other features of cognition, such as whether speakers possess cognition (Potter and Edwards, 2013, p. 704). Sacks’ argument has been well supported by the CA community, noted for its agnostic stance toward cognition (Hopper, 2005). Yet Enfield and Levinson (2006, p. 7) challenge this state of harmony: “But they [conversation analysts] are equally interested in intersubjectivity, the way in which a shared understanding is arrived at.” Overall, what Merritt (2021) suggests about the intertwining of intersubjectivity and cognition aligns with the stance taken by this study.

Drew (2005) and Potter and Edwards (2013) found that cognition can be inferred from interaction (e.g., an analyst from a transcript), but participants do not always topicalize cognition in their talk. Potter (2006) criticizes attempts to impose cognitivist assumptions on CA. However, Drew also found that a participant’s confusion in conversation is something that needs to be explicitly resolved by the participants. Importantly, “a source of cognition” is, therefore, interaction (Drew, 2005, p. 181) or intersubjectivity in interaction (Merritt, 2021, p. 356). These interaction perspectives on cognition can provide fresh alternatives to animal cognition studies (e.g., Willgohs et al., 2023), which often seek “underlying cognitive structures, mental processes or neuronal objects” (Potter and Edwards, 2013, p. 704). At best, this article can serve as a bridge between interspecies pragmatics and animal cognition studies.

An anthropomorphic but important theory is the “human interaction engine” (Levinson, 2006a). The engine addresses cognitive properties in human interaction (after Levinson, 2006b, p. 87): (1) mind-reading abilities (for example, knowing that you know what I know), (2) reflexive mind-reading abilities (for example, knowing that you know what I want you to do and that you are aware that I know what you want to do), (3) the capacity to understand Gricean intentions (for example, understand intentions in conversation), and (4) ethologically grounded practices (e.g., rituals for greetings; multimodality in communication; the drive for seeking cooperation). While prior studies have criticized the engine for not being designed for non-human animals (Logue and Stivers, 2012; Rossano, 2013), recent studies have provided some evidence of non-human animals’ participation in the engine, e.g., in greetings (Nilsson and Norrthon, 2024). However, Levinson (2006a, p. 55) suggests that “ethological patterns” such as turn-taking may exist without this engine. This study relates to the question of whether the engine is beyond the reach of the non-human animals. At the outset, it seems that Gricean intentions were meant to apply only to conversation.

While the focus of this paper is on human–dog ball games, dogs’ participation in meaning-making has been investigated in other contexts as well. Ethnographic observations conducted within an assistance dog agency show that dogs engaged in assistance work were understood as making meaning through their free choices (Edminster, 2011). Based on these choices, agency staff framed the dogs using different “career” labels: “The dogs’ careers are characterized by independence, free choice, and personal reward” (Edminster, 2011, p. 135). Importantly, the dogs’ careers were seen as indicating their position within the agency. For the purposes of this study, these findings illustrate how assistance dogs participate in the meaning-making processes within the institutional context. In this regard, this study also addresses meaning-making processes in everyday context.

Interspecies pragmatics research based on CA can provide systematic findings from human engagement with non-human animals. Indeed, Morris (1938) argued that pragmatics considers the biotic aspects of semiosis (i.e., meaning-making):

Since most, if not, all, signs have as their interpreters living organisms, it is a sufficiently accurate characterization of pragmatics to say that it deals with the biotic aspects of semiosis, that is, with all the psychological, biological, and sociological phenomena which occur in the functioning of signs.

(Morris, 1938, p. 108)

Morris (1938, p. 81) describes semiosis as the process “in which something functions as a sign.” This understanding of semiosis includes components that can be summarized simply as a rule: “a sign refers to something for someone.” During the 1960s, those ideas spearheaded a new branch of research in pragmatics known as zoosemiotics, defined as “[t]he science of signs … devoted to the scientific study of signaling behavior in and across animal species” (Sebeok, 1967, p. 81). For example, dancing bees were found to inform other bees (Sebeok, 1967). Thus, non-human animals were considered “someones” for the purposes of semiosis. Today’s perspective calls for contributions to interspecies pragmatics; a limitation of earlier studies on pragmatics is that they primarily focused on semiosis, either among humans or “in and across” various animal species. Therefore, this study contributes to interspecies pragmatics by considering humans and non-human animals in discovering the biotic aspects of semiosis occurring in interaction. Moreover, as noted earlier, this study introduces cognitive perspectives to interspecies pragmatics, which have been lacking in the field. Meaning-making processes are grounded in the interaction where semiosis occurs in turn-taking between interspecies participants. For example, Saalasti et al. (2023, p. 324) suggest that meaning-making processes are related to repair activities. We expand this suggestion based on the findings of this article.

## 3 Data and methods

This research is part of the Human-Dog Interaction Study (2017–2020), which aimed to produce new information on interspecies interaction, provide evidence for shared understanding between the two species, and investigate the social actions that humans and dogs use to maintain their interaction. For the study, we collected videotaped data from everyday interactions, as well as experimental and medical interactions. The data for this sub-study were collected in collaboration with a dog owner in southern Finland over 5 days during the winter of 2017 as part of everyday data collection. Research permission was obtained from the dog owner prior to the launch of the sub-study.

The dog owner was recruited to equip her dogs with harnesses that enabled GoPro video cameras to be attached to their fronts. The video recordings provided a first-person view based on the direction in which the dog was positioned. The resulting footage, totaling around 2 h (approximately 52 GB), was transcribed using ELAN 7.0 and analyzed through CA (Sidnell and Stivers, 2013). During the 5 days of recording, two German shepherd dogs and one Belgian Shepherd (Malinois) organized their interactions with the dog owner. While the data have been transcribed following Jefferson’s conventions (Jefferson, 2004), our focus lines are transcribed using conventions developed by Simonen (2023) to address interspecies interactions, where milliseconds better capture rapid animal actions. In addition to animal actions, milliseconds are also used with human actions in our focus lines (see Supplementary material for transcription conventions).

The setting has been described in more detail in earlier publications, and the footage has been analyzed previously. Prior studies focused on dogs’ embodied responses to human turn-taking (Simonen, 2023) and interspecies group dynamics (Simonen, 2021). Those studies found that pragmatic meaning is a co-produced gestalt of a ball-throwing and catching exercise (Simonen, 2023, p. 70). For this study, the footage reveals walking sessions in which dogs offer their balls to the owner, receive their balls, and attempt to repair the human’s unexpected actions. The events captured in the footage involve human and animal actions that serve as prime examples of interspecies pragmatics, highlighting the role of animal actions in human meaning-making processes. In this way, the study provides additional granularity regarding the earlier interpretation of pragmatic meaning by identifying the actions behind the gestalt.

## 4 Analysis

In this section, we analyze our data to provide empirical evidence of the meaningful behavior that informs our pragmatic analysis. First, we investigate how the dog initiates a ball-catching exercise by offering its ball to the human and how the human responds to this offer. We argue that these actions establish a pre-sequence: “summons” and “answer.” Schegloff’s (1986, p. 130) concept of two-turn and three-turn packages has been further developed to include embodied forms of actions within these packages. Next, we consider the main sequence where the human delivers the ball back to the dog and the dog receives the ball: “deliver the object” and “receive the object.” Finally, we identify one case from the data where the human displays less meaningful behavior toward the dog’s offer. In that case, the dog uses repair initiators to address the human’s unexpected actions. As we can see, the human rapidly repairs their mischief. Overall, the analysis provides empirical evidence of animal actions’ involvement in the human meaning-making processes in interaction.

### 4.1 Pre-sequence: dog summons the human and the human answers

**Figure 1:**
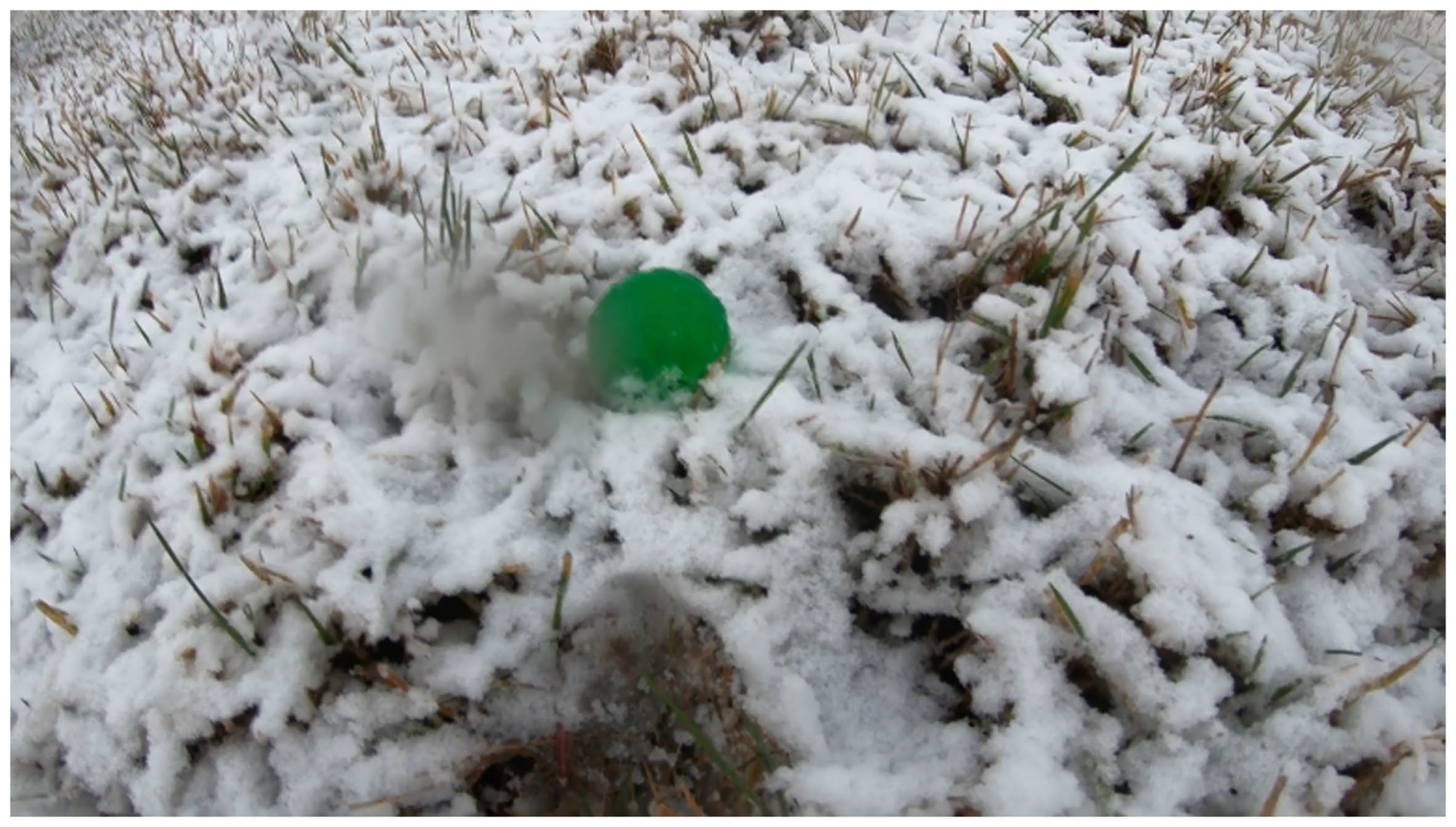
The dog has dropped the ball.

This section analyses pre-sequences in ball games in human-dog interaction. Pre-sequences, or pre-extensions (Stivers, 2013, pp. 193–196), often precede the main sequence. Common to different types of pre-sequences, such as pre-announcements, pre-requests and pre-tellings, is their role in setting up expectations for the main sequence. In the analysis, it is the dog who initiates the pre-sequences (unlike the common situation in which a human summons the dog). Extracts (1–3) consider events where the dog offers its ball to the human, and the human either picks the ball up from the snow (1–2) or prepares the playground instead (3). When we examine the event described in Extract (1), we find the dog (DOG) waiting nearly 2 s before picking up its ball (BAL) from the snow (lines 1–2; Figure 1). While the ball’s agency represents a non-human actor in interaction, its representation is outside the scope of this study.

**Extract (1) (04:28–04:48)** 
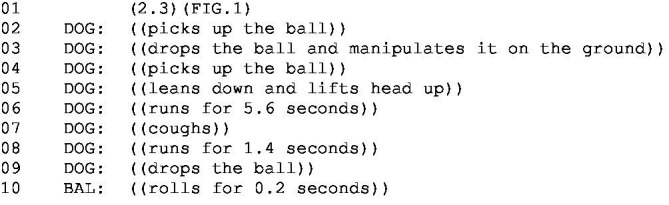


This extract is transcribed according to the conventions of Jefferson (2004). Line 1 indicates the time in seconds (nothing happens; see Figure 1), while the rest of the lines contain the transcriber’s comments. Although the comments were initially intended to provide additional information about the conversation, they also reveal the stepwise progression of the interaction. From the comments, we find that after the dog picks up and drops the ball, it manipulates the ball again before picking it up (lines 2–4). With the ball in its mouth, the dog stretches and begins to run; during this, it coughs (lines 5–8). Finally, the dog stops and drops the ball, which rolls in the snow (lines 9–10). Line 9 is considered the first position in this pre-sequence.

**Figure 2:**
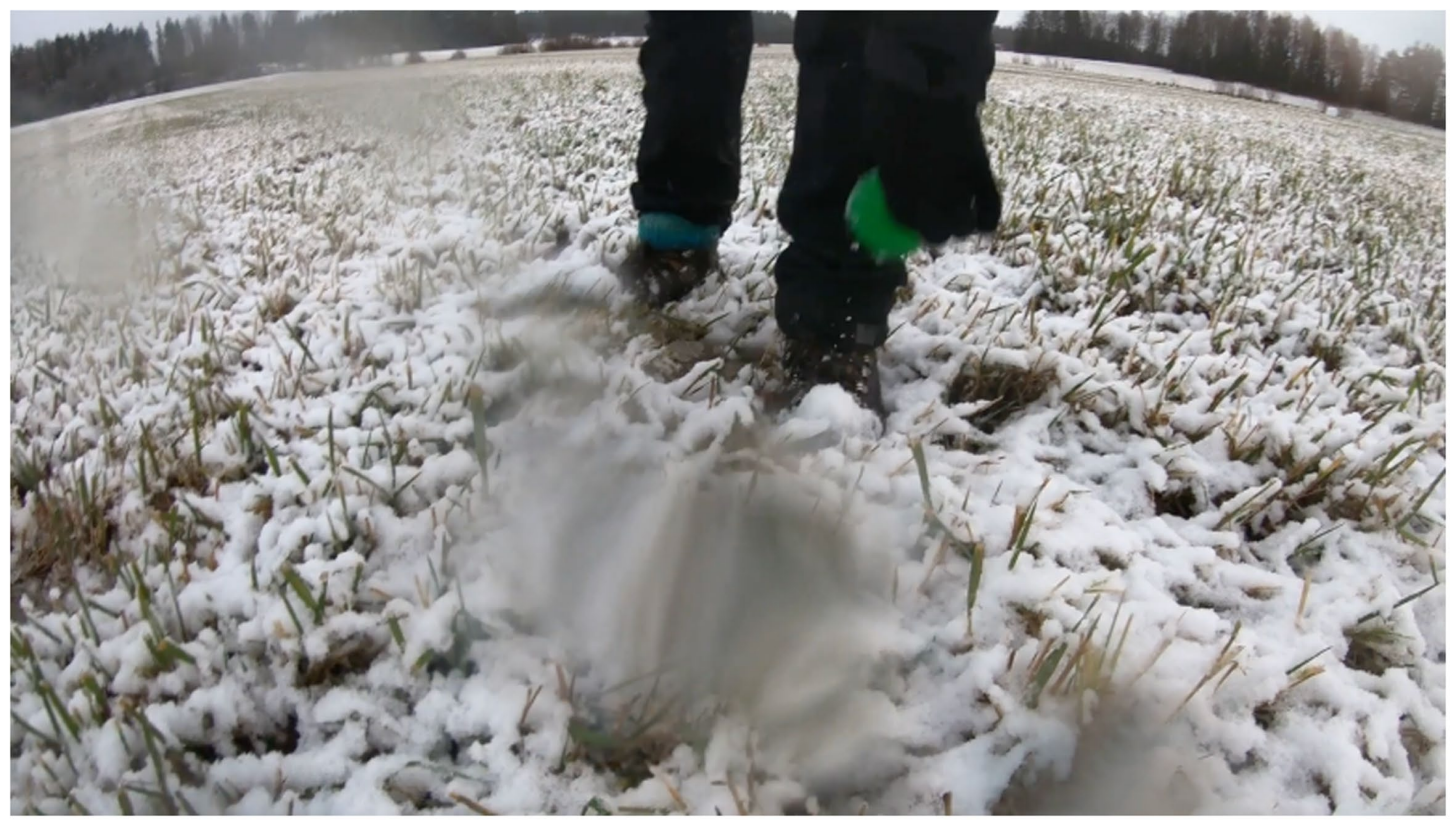
The dog begins to run after the human has lifted the ball for 110 ms.

Next, the human arrives from the direction behind the waiting dog (lines 11–12). Note that focus line 18 is transcribed in more detail than the other lines. This focus line shows the progression of the interaction in milliseconds and is read from left to right. Plus signs (“+”) divide turns of talk and embodied actions into analytic segments, while minus signs (“−”) indicate actions that continue across analytic segments. Figures are indicated with a hashtag (“#”) followed by the figure number. The location of the hashtag within an analytic segment (e.g., on line 18, the hashtag is at the end of the segment) shows the position of the figure in relation to the duration of the segment. On line 18, Figure 2 is located at the end of the analytic segment, which lasts for 110 ms. Arrows (“–>”) refer to actions that continue across the lines of the transcript. Embodied actions, balls and figures are described using uppercase letters (HUM, DOG, BAL and FIG), while speech is labeled with lowercase letters (hum).


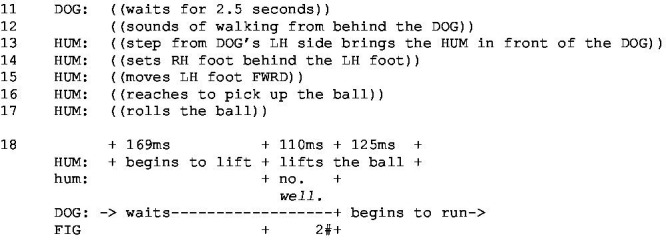


The human approaches the dog from the left side and positions themselves so that the ball is situated between them (lines 13–15). From this location, the human reaches to grab the ball; however before picking it up, she rolls the ball in the snow (lines 16–17). In focus line 18, we observe that as soon as the human lifts the ball and says *no* (“well”), the dog begins to run. It takes only 110 ms for the dog to recognize that the human has received the ball allowing the dog to start running (Figure 2). According to Vepsäläinen (2019, p. 69), the particle *no* is often used to signal transitions. In this context, the particle indicates a sequential transition from the second position to the third position; the dog begins to run immediately after the particle is uttered and the ball is lifted.

As mentioned above, this section focuses on pre-sequences leading to the main sequence. For this purpose, lines 2–8 represent the means by which the dog prepares, culminating in line 9, where it deploys a “summons” that recruits the human. Lines 12–17 detail the human’s preparatory work, and line 18 indicates to the dog that the human has finally provided an “answer.” Together, these actions constitute an adjacency pair involving the animal action of “summons” and the human action of an “answer.” Clift (2016, p. 77) writes: “The summons-answer adjacency pair, … is a generic presequence, whereby one speaker recruits another to interact by securing their attention.”

Extract (2) shows a similar sequential development to Extract (1). Here, the dog works with the ball and its body before starting to run with the ball in its mouth (lines 1–6).

**Extract (2) (01:00–01:21)** 
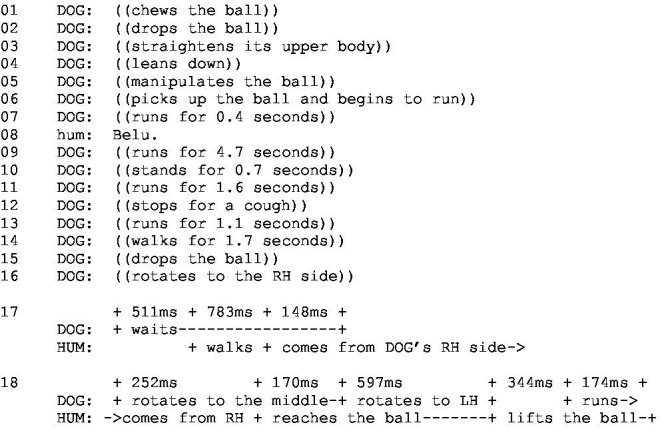


The human calls the dog by name right after the dog starts running (lines 7–8). The dog continues running and, after 4.7 s, stops briefly (lines 9–10). Another stop occurs when the dog pauses to cough (line 12). Finally, in lines 15–16, the dog drops the ball and orients itself by rotating its body to the right-hand side. Focus lines 17–18 indicate that the human approaches from the right-hand side; thus, the dog was expecting the human to arrive from that side. After 344 ms of the lifting of the ball, the dog behaves as if it has gained the human’s attention and begins to run, as in Extract (1).

In Extracts (1–2, line 18), the dog secures the human’s attention and prompts her to pick up the ball. The dog, beginning to run, is certain that the human knows it wants her to throw the ball. In terms of Levinson’s engine (Levinson, 2006b), this is treated as a form of mind-reading in human communication. From an interspecies pragmatic point of view, animal actions are involved in human meaning-making processes if the human indeed throws the ball. In section 4.2, we find that the human knew what the dog wanted and was aware that the dog knew what she knew when she proceeded to the main sequence. This is known as reflexive mind-reading in Levinson’s engine (Levinson, 2006b). These cognitive operations found in human conversations seem to occur in human-dog interactions.

Extract (3) begins with the dog running and dropping the ball (lines 1–2). This case demonstrates that the dog’s summons action does not lead to a single outcome. Here, the human does not pick up the ball; the dog waits while the human prepares the playing area. Does the dog understand what the preparation entails? One can observe that an agreement with the dog’s agenda is shown through careful preparations around the ball. Therefore, it seems that the human’s embodied actions sequentially unfold, leading up to a closing of the pre-sequence while keeping the recipient’s attention engaged. Such “embodied storytelling” fosters intersubjectivity between the participants.

**Extract (3) (02:15–02:34)** 
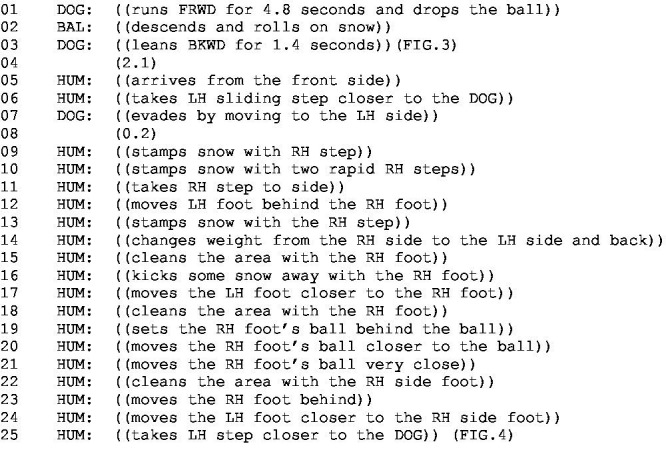


**Figure 3:**
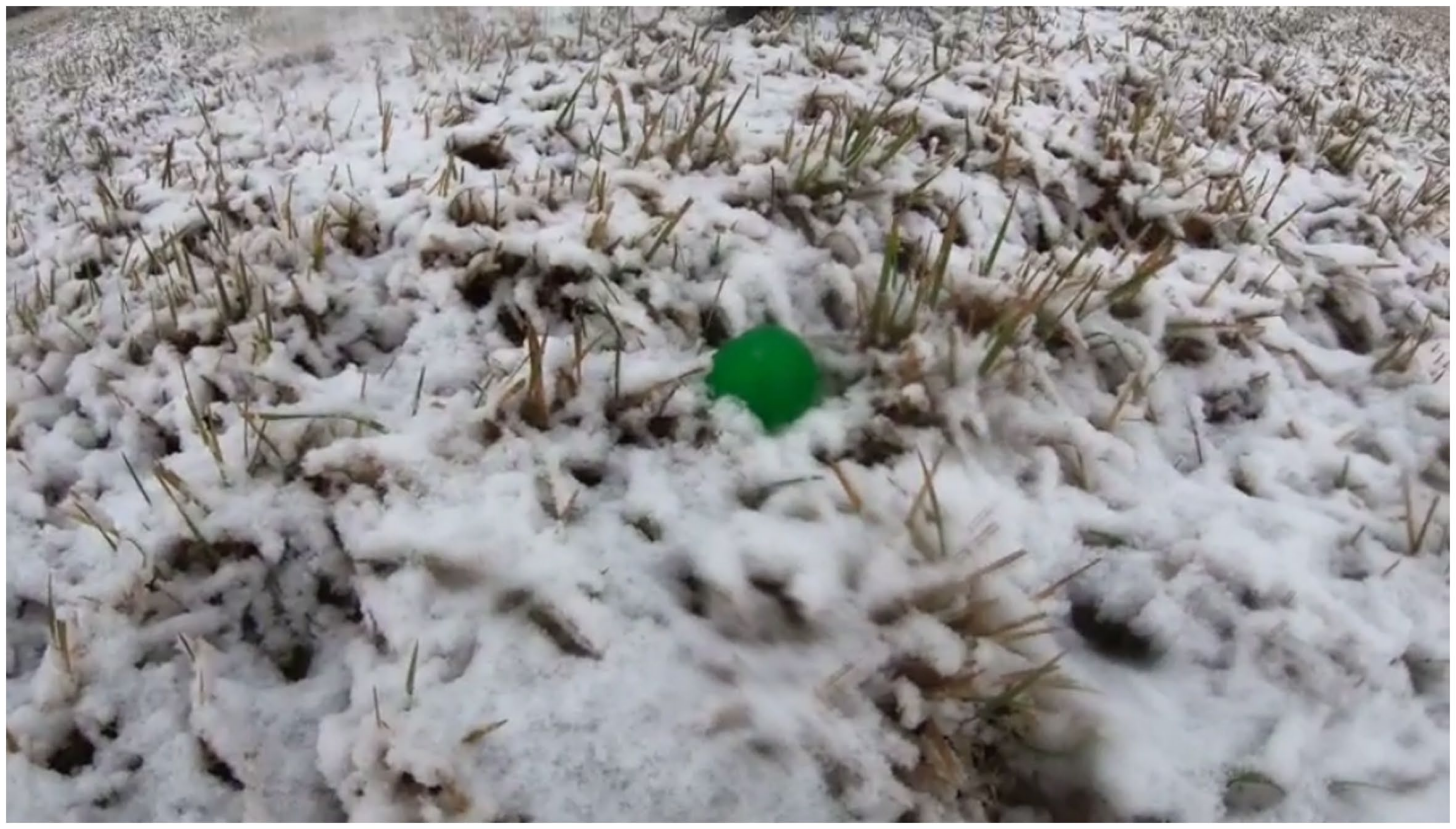
The dog’s right paw has left a trail in the snow as the dog leans back.

**Figure 4:**
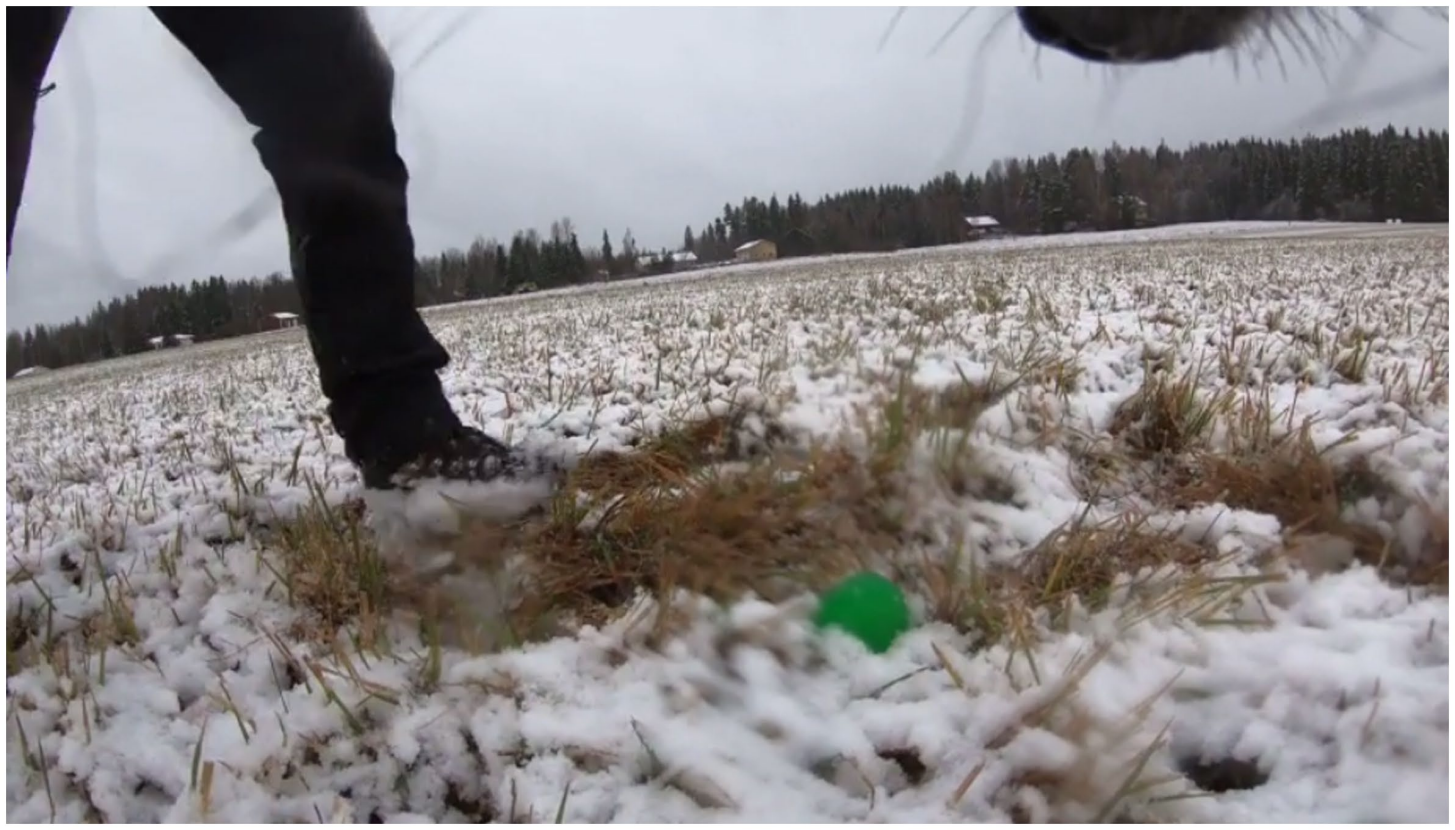
The human has cleared the playground without lifting the ball.

The above pre-sequence differs from the previous cases analyzed in this section, as the dog leans backward after letting the ball drop (lines 2–3; Figure 3). From that position, the dog awaits the human, who is approaching directly from the dog’s front side and nearly sliding over the ball and the dog (line 5–6). The dog evades the sliding foot by moving to the left (line 7) and then waits for the human until the end of this pre-sequence. As it stands, the human does not pick up the ball but instead begins to prepare the snowy ground for kicking the ball. This involves stamping snow (lines 9–10, 13), changing foot positions (lines 11–12, 14, 17, 23–25), clearing the area (lines 15, 18, 22) and testing a suitable distance for kicking the ball (lines 19–21). After these efforts, the playing area is ready (Figure 4), and the pre-sequence is complete.

Overall, this section considered pre-sequences, and the cases presented indicate that deploying a “summons” through preparatory work (e.g., running, walking and waiting) and finally dropping the ball elicits “answers” that demonstrate agreement with the summoner’s initiative: the recipient either picks up the ball or begins to prepare a playground. We found that in interspecies interaction, two-turn and three-turn packages concerning “summons” can be achieved through embodied actions, while “answers” are delivered using a combination of turns of talk and embodied actions. Extracts (1–2) demonstrate the structure: summons-answer-third position closing. Specific to this structure, the third position closing turn is performed by the summoner, who then runs away. Extract (3) focuses on the summons-answer structure, in which the dog remains in place. In addition, this section considered mind-reading and reflexive mind-reading in human-dog interaction. These analyses correspond to discussions in human interaction about whether an analyst perceives cognition while human participants do not orient themselves to it (e.g., Potter, 2006). Generally, participants organizing a pre-sequence do not address cognition; however, the analyst can observe how their interaction is organized and what outcomes the pre-sequence entails. Following this, the analyst can demonstrate the relationship between the pre-sequence and the main sequence, indicating how occurrences in the pre-sequence materialize in the main sequence.

### 4.2 The main sequence: the human delivers the ball and the dog receives it

This section contains Extracts (4–6), which demonstrate the main sequence of the ball games between humans and dogs. Thus, the main sequence can be initiated without any pre-sequences if, for example, the human brings the ball to the game and the participants establish a shared understanding of the upcoming ball game. In our case, the dog initiates the game by summoning the human to play and thereby setting expectations as the owner of the agenda. First, Extracts (4–5) show how the human continues the interaction after the pre-sequence by throwing the ball, and the dog responds by catching it. In Extract (6), the human continues the interaction by kicking the ball, and the dog responds by catching it.

Extract (4) continues from Extract (1). At the end of the pre-sequence, the dog starts to run away from the human, who picks up the dog’s ball from the snow. We arrive at the scene about 10 s later and find that the dog is running forward while looking to the side and backward before catching the ball. Unfortunately, we do not see in the video footage when the human throws the ball.

**Extract (4) (04:58–05:05)** 
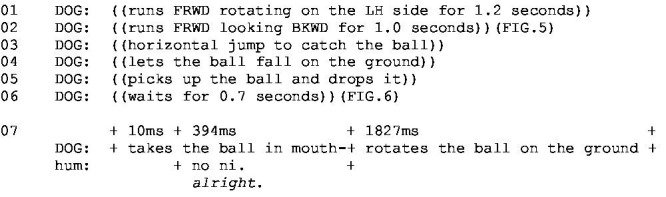


**Figure 5:**
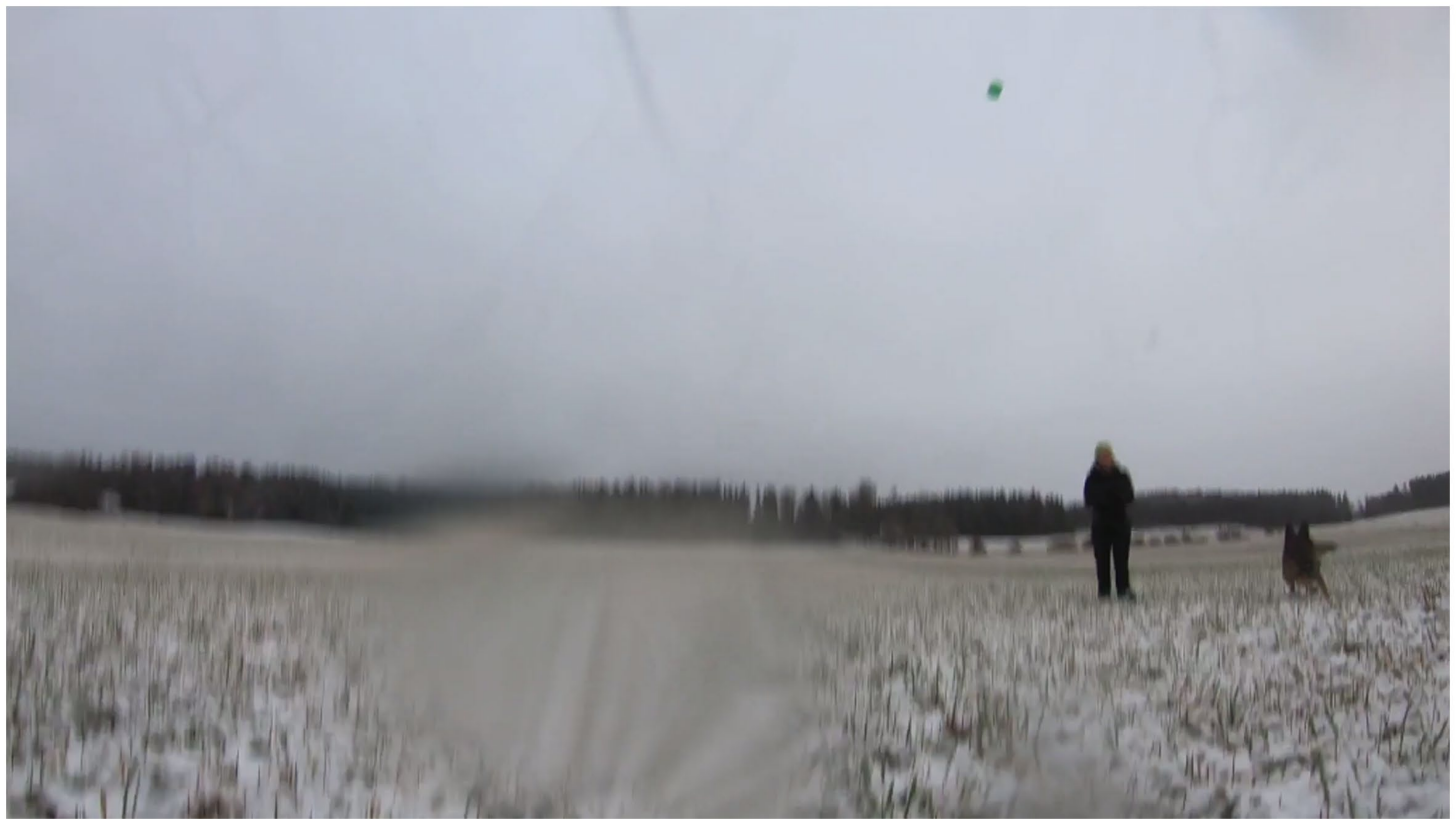
The dog looks behind and observes that the human has thrown the ball.

**Figure 6:**
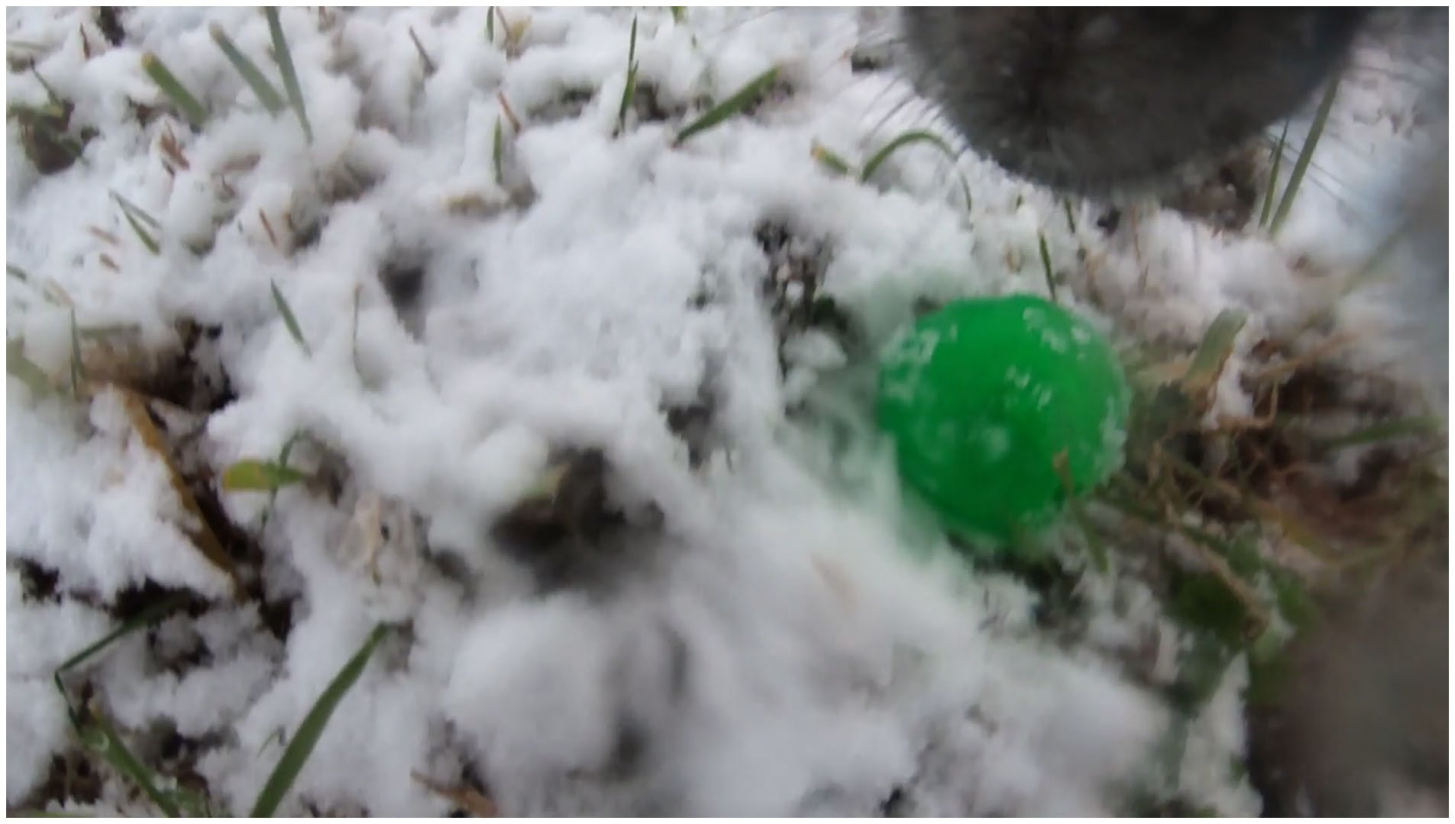
The dog has received the ball and then dropped it in the snow.

When the dog looks backward (line 2; Figure 5), the human is visible but has already thrown the ball, which constitutes the first position of the main sequence. In line 3, after looking backward for 1 s, the dog turns around, jumps horizontally, and catches the ball, thereby performing the second position. The dog then lets the ball fall onto the snow (line 4). After picking up and dropping the ball again (line 5), the dog pauses briefly (Figure 6) and, in focus line 7, takes the ball in its mouth as the human produces *no* *ni* (“alright”), thereby implementing a sequence-closing third. Immediately following the human’s turn, the dog begins to play with the ball. At this point, the main sequence is now closed, and the dog has regained possession of the ball. Vepsäläinen (2019, p. 87) reports that the particle *no ni* is commonly used in preparing to end a phone call; here, it indexes the approaching closure of the main sequence.

To recap, in this main sequence, the first position is occupied by the human’s throwing of the ball, the second by the dog’s catching of it, and the third, which closes the sequence, by the human’s production of a particle.

The pre-sequence for Extract (5) was shown in Extract (2). There, the human called the running dog by name. When we return to this scene, we find that 12 s have passed since Extract (2). Now, the dog is lying on the snowy ground and watching the human. In focus line 4, the human calls again. The dog gets up and begins to run only 256 ms after its name has been uttered.

**Extract (5) (01:33–01:44)** 
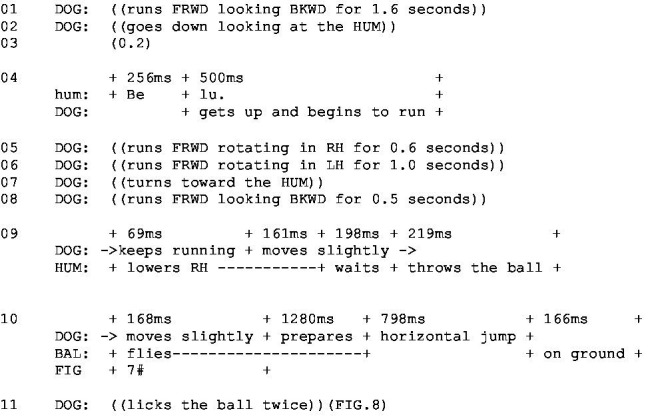


**Figure 7:**
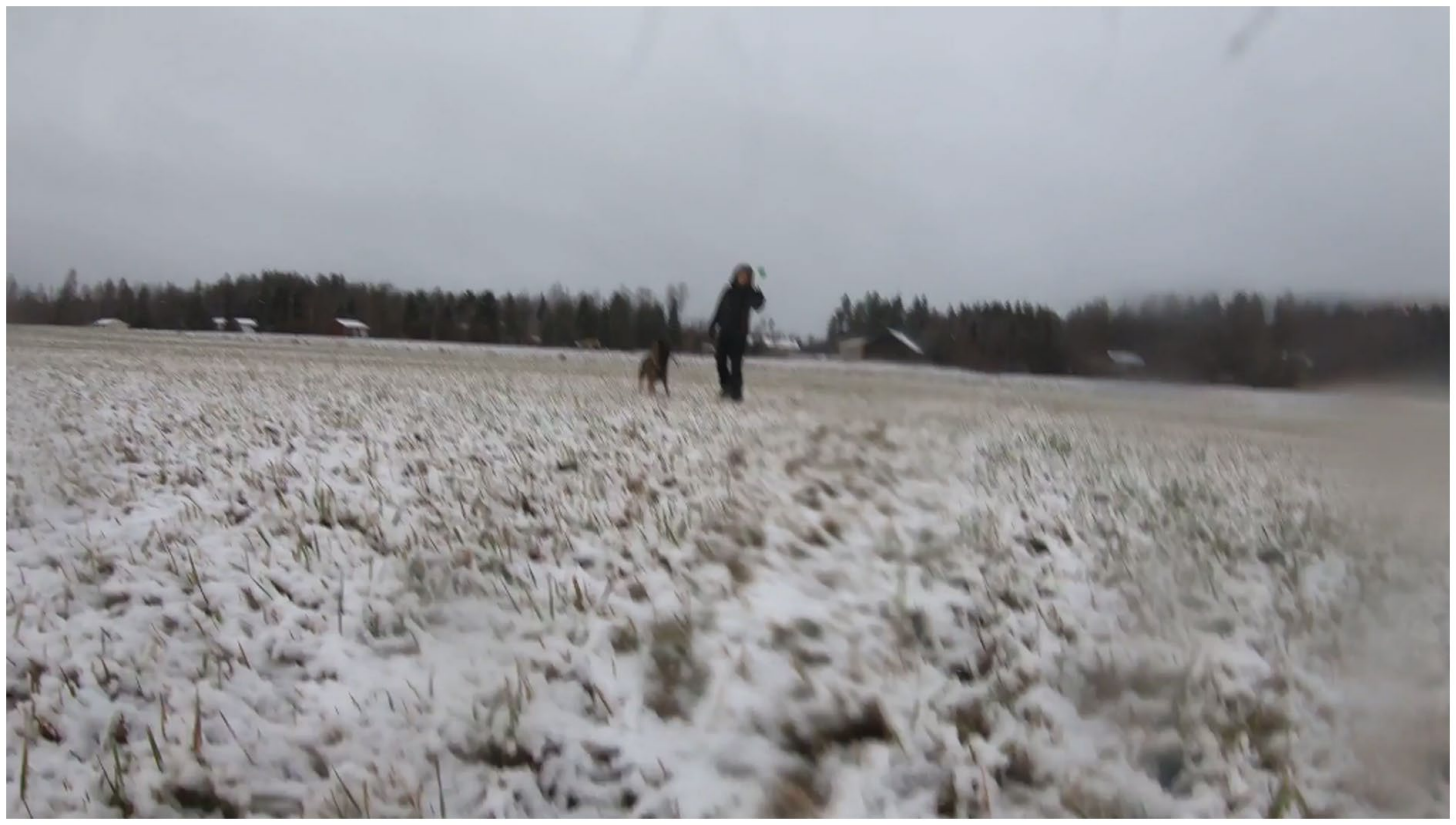
The human has thrown the ball.

**Figure 8:**
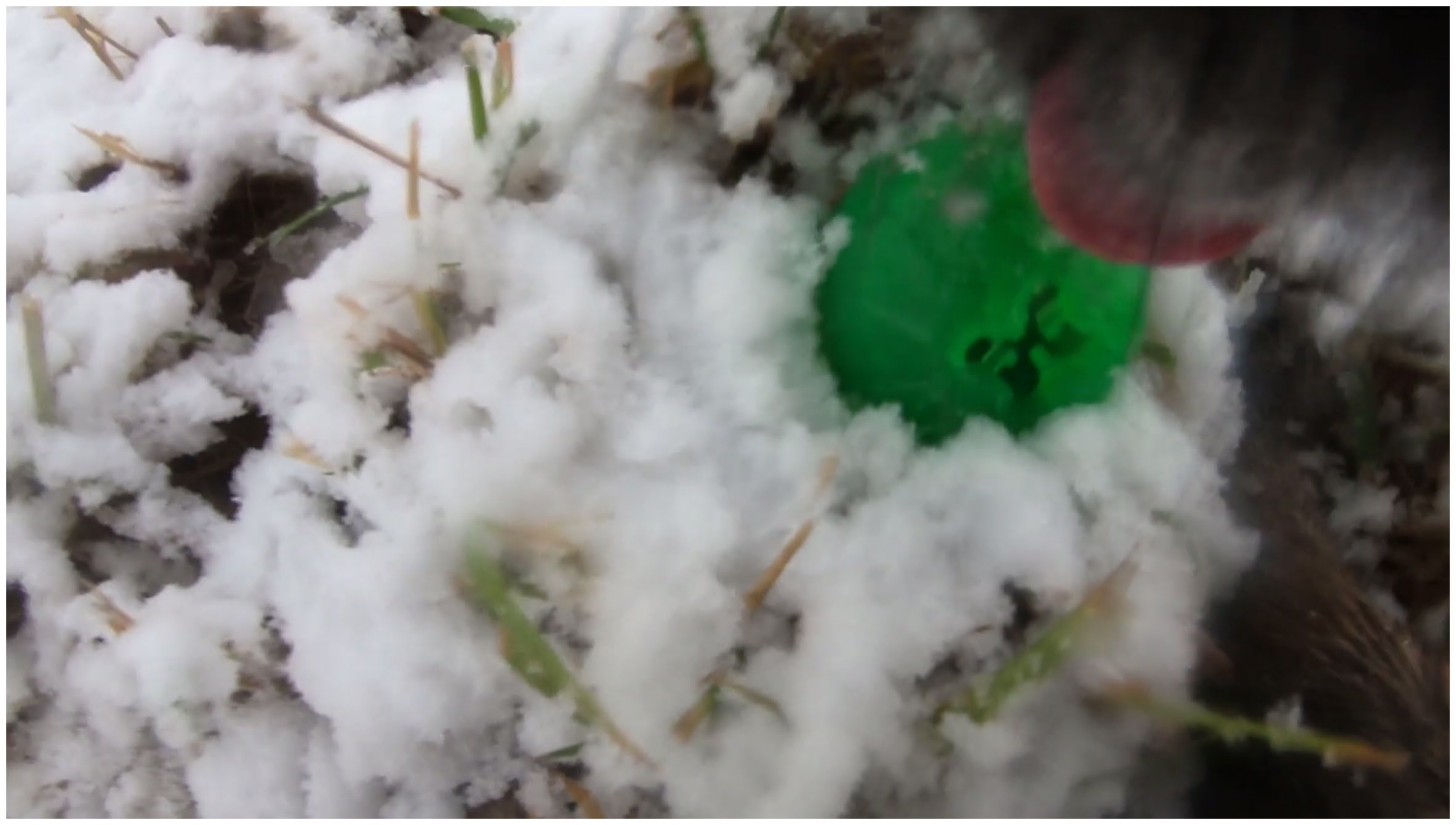
The dog has received the ball and is now licking it.

The dog runs away from the human (lines 5–8), who is holding their right hand up high. Focus line 9 shows that the human lowers their right hand and waits for 198 ms before throwing the ball. In this interaction, lowering the hand signals the upcoming throw. Simultaneously, the dog observes the human while moving away (Figure 7). Line 10 is also a focus line, where the ball flies for 1,448 ms before the dog catches it. Again, the dog makes a horizontal jump. In line 11, the dog has gotten its ball back and licks it (Figure 8).

So far, we have analyzed two main sequences. Extract (5) concerns a shorter two-turn package: “deliver the object” and “receive the object.” Extract (4) involves a longer three-turn package: “deliver the object,” “receive the object,” and “acknowledge that the sequence is coming to a close.” The interspecies partners us embodied practices of “throwing the object” and “catching it by jumping horizontally.”

Our last case in this section is Extract (6), which is a follow-up to the pre-sequence shown in Extract (3). This pre-sequence differs from the other pre-sequences in that the human did not take the dog’s ball but prepared the snowy ground in the front of the dog, so that it would be suitable for kicking the ball.

**Extract (6) (02:34–02:44)** 
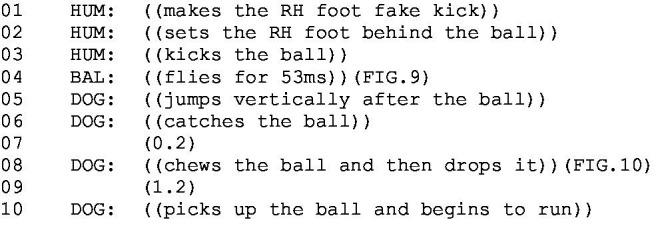


**Figure 9:**
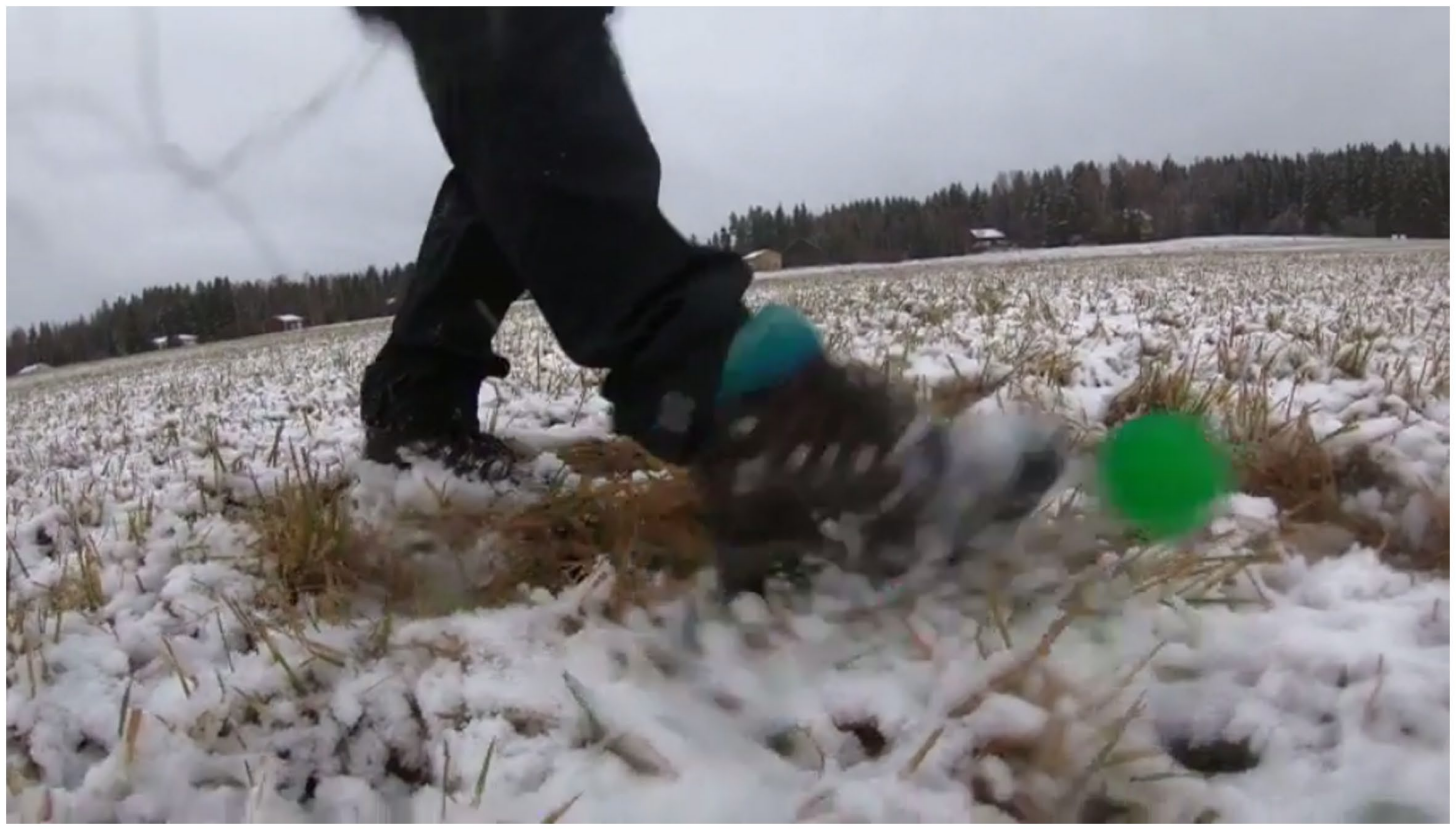
The human has kicked the ball.

**Figure 10:**
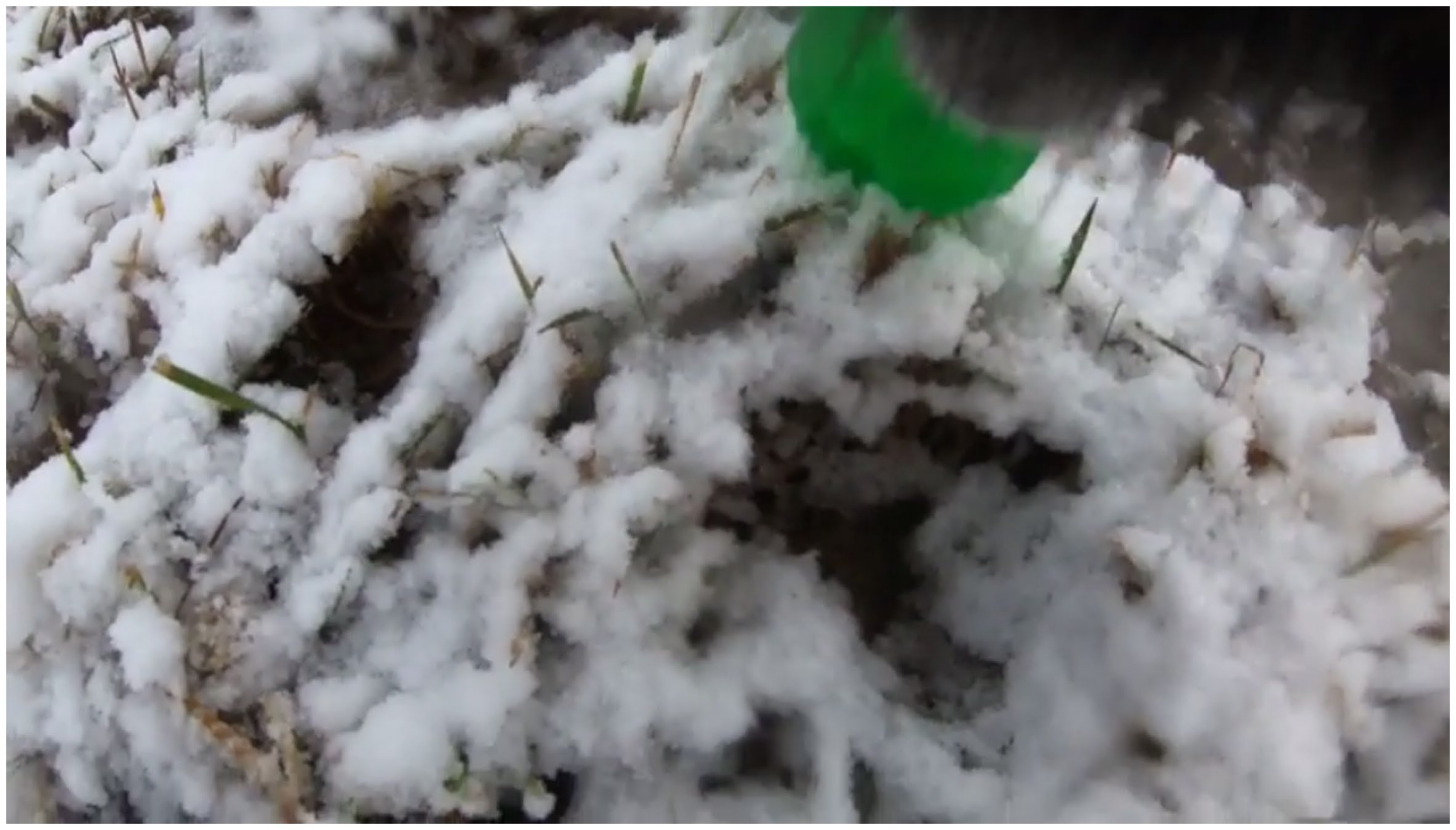
The dog has received the ball and then dropped it in the snow.

The human makes one fake kick and then positions their foot behind the ball (lines 1–2). The kick is done with the tip of the shoe (line 3) and the ball flies only 53 ms (Figure 9). The dog is nearby and jumps vertically to catch the ball (lines 5–6). After regaining its position, the dog chews the ball and then drops it (Figure 10). The main sequence is complete, and the dog takes the ball and runs (line 10).

To summarize, this section examined the adjacency pair of the main sequence: “deliver the object” and “receive the object.” There were alternatives for the practices used to perform these actions: “throw/kick the object” and “catch by jumping horizontally/vertically.” Thus, the main sequence was structurally a two-turn package in Extracts (5–6). Extract (4) was concluded with the human’s third position turn, indexing the approaching end of the sequence. In fact, the dog was not yet ready to move on but started playing with the ball when the utterance arrived. From an interspecies pragmatics perspective, this section provided evidence of the human’s understanding of what the dog wanted her to do in the pre-sequences: the dog brought expectations for her to throw or kick the ball. Humans often organize ball game exercises for the dog, reversing expectations. In Extracts (4–5), we saw how the human completed the dog’s agenda by throwing the ball, which the dog then caught. Extract (6) showed that kicking the ball is an alternative to throwing.

### 4.3 The repair organization: the dog deploys repair initiators due to the human’s actions

This section contains only one case, but it suggests that the repair organization (Schegloff et al., 1977) initially found in human conversations is shared with dogs as well. Repair organization concerns troubles in speaking, hearing, or understanding (e.g., Kitzinger, 2013). Saalasti et al. (2023) in their scoping review of embodied practices used in repair during human-to-human interaction, found that the human body (e.g., eyes, face, head, hands, and feet) can be configurated in different ways to indicate (the need for) repair. These configurations of multiple body parts seem to cluster: “The theoretical implications of this clustering have yet to be explored fully, but we can suggest that, when it comes to the body, at least in the domain of repair, the constraints on meaning-making semiotic resources are relaxed, and so there may be no limit on the semiotic resources used” (Saalasti et al., 2023, p. 324). Where Saalasti et al. (2023) based their findings on repairing in human conversation, this study attends to human-dog interaction. When doing so, the theoretical implication of the clustering of dog body parts receives some contribution.

Goode (2007) gives an example of a game move that is problematic for his dog Katie. We might wonder: why is Goode doing this? Schegloff et al. (1977, p. 381) raise the possibility that repairing has socialization purposes. While Schegloff et al. considered adult-child interaction, we expand the range to include human-dog interactions, where socialization is likely to happen. Goode describes (Goode, 2007, p. 33):

Toward end of the sequence, just before striking the ball, I place the ball under my foot and control it there, moving my foot back and forth. This is a particularly problematic move for Katie to react to because the possibility exists that I could kick it any direction from that position. … The particular kick I use employs the knowledge that “ball under the foot” is a game move which Katie orients, and finds problematic.

In the following, the human deploys the “ball under the foot” game move. The pre-sequence begins similarly as other analyzed extracts: the dog is running and playing with the ball until drops it (lines 1–8). For the pragmatic analysis, this extract serves as a deviant case, as the dog may momentarily find the human’s behavior to be meaningless.

**Extract (7) (00:26–00:54)** 
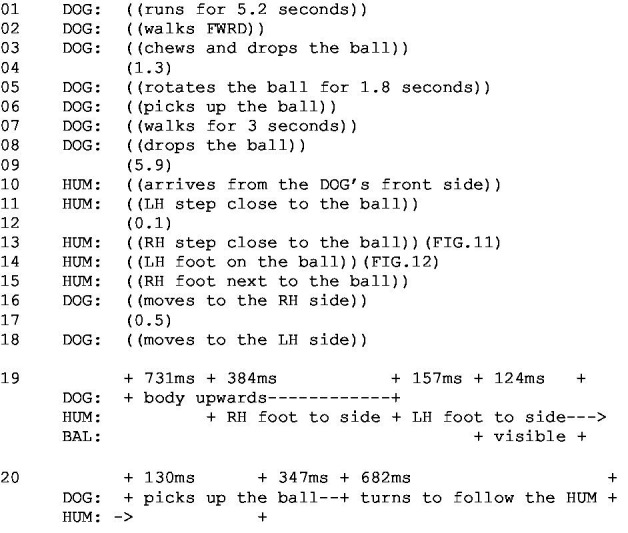


**Figure 11:**
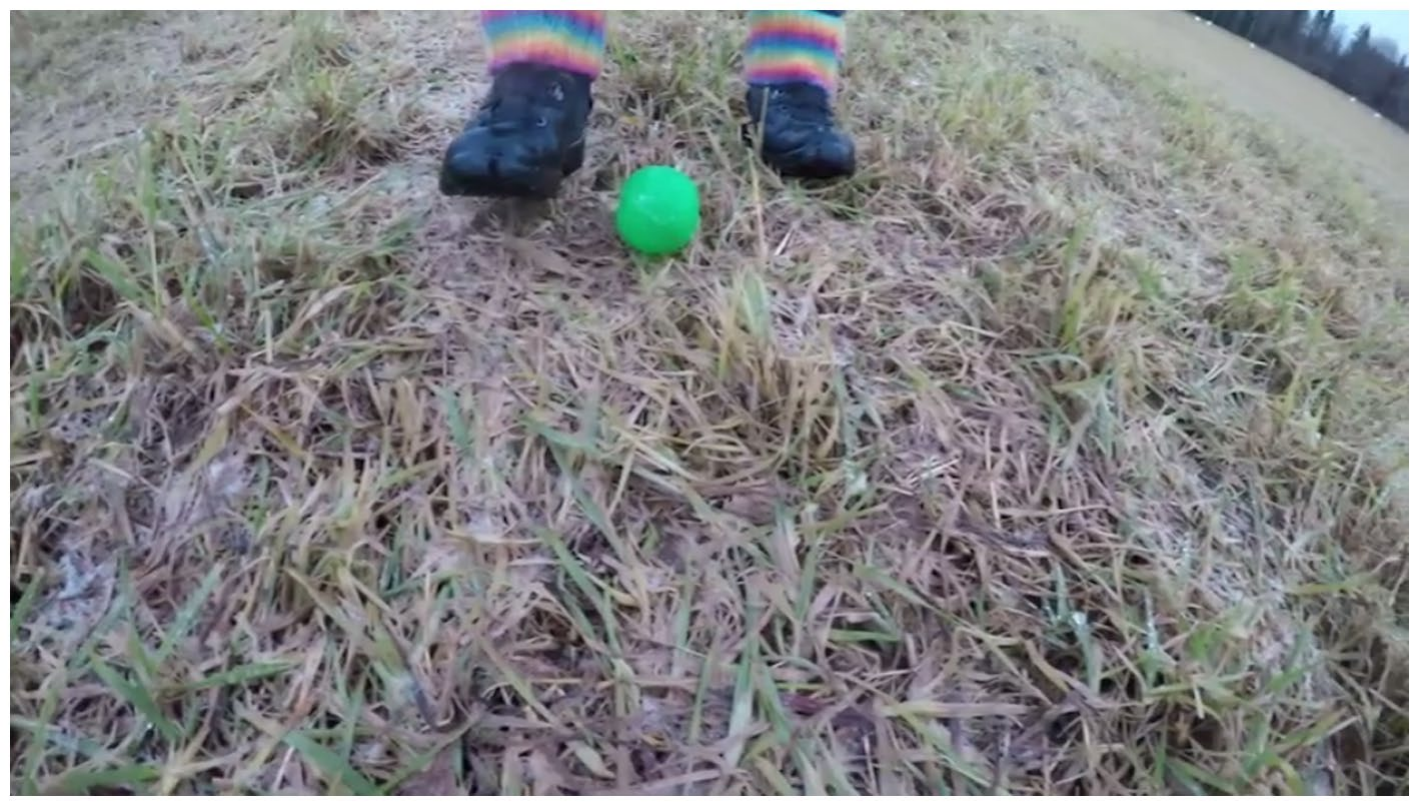
The human comes close to the ball.

**Figure 12:**
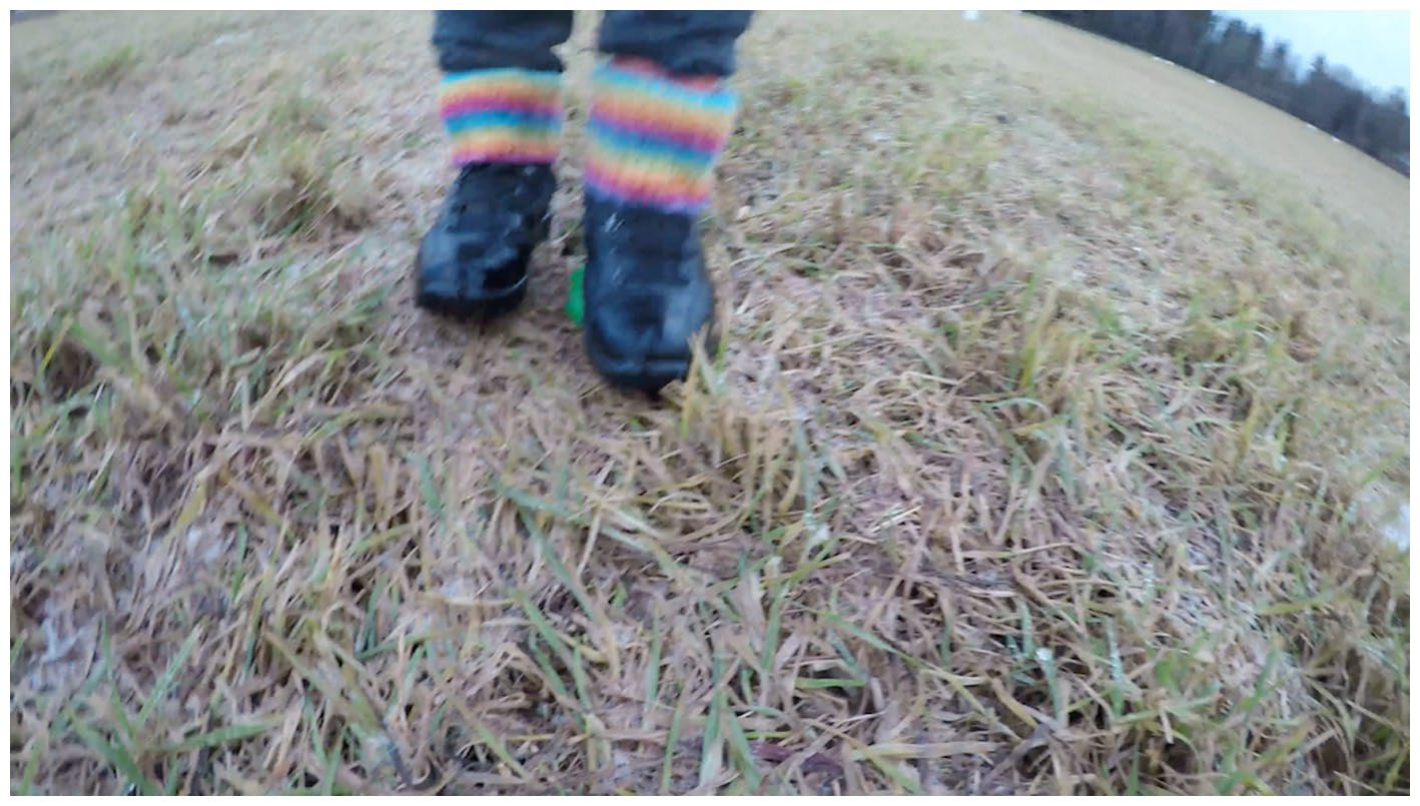
The human steps on the ball.

The dog waits nearly 6 s before the human comes close to the ball (lines 9–13; Figure 11). Then, in line 14, the human steps with their left foot on the ball, which is no longer visible (Figure 12). After that, the human aligns their feet (line 15). The dog makes two additional attempts of repair. First, the dog attempts to repair the situation by moving its body slightly to the right (i.e., toward the side of the human’s left foot), then waits for 0.5 s before moving to the left, bringing its body back to the center (lines 16–18). The first attempt does not achieve the intended solution.

Focus lines 19–20 show that the dog lifts its front body upward (i.e., the dog looks at the human’s face). This embodied action takes 1,115 ms and serves as the second initiation of repair, attempting to correct the human’s misconduct. It needs only 731 ms from the beginning of the dog’s repair initiator until the human begins to address their misconduct by moving away. First, the human takes a right-foot step to the right, rotates their body, and takes another step, which reveals the ball. It takes only 124 ms for the dog to react and pick up the ball. Then, the dog follows the human by running after her. If the dog had a misunderstanding about the human’s odd behavior, no hard feelings were displayed at the end of the sequence.

Extract (7) begins with a “summons,” but it does not catch the recipient’s attention with the summoner’s agenda. The dog does not receive such an answer that would lead to the main sequence of the ball-catching exercise. The dog eventually receives its ball after the failed summons episode.

In this section, we analyzed the pre-sequence where the dog attempts to repair unexpected human actions. Previously, it was stated that in these pre-sequences, the dog is the owner of the agenda when the dog initiates the sequence; the dog has expectations on how humans should play; these expectations are the sign for the recipient to play the game. We found that in pre-sequences the precise moment when the dog can be sure the human knows that the dog wants her to throw a ball is at the core of intersubjective understanding between the human and the dog. Then, this section added that we can treat intersubjectivity as “a source of cognition” (Merritt, 2021, p. 356) where the dog is not different from humans in the interaction and the sequence organization when they use repair organization to maintain intersubjectivity. Drew investigated human interaction and found (Drew, 2005, p. 181): “The action (the repair initiation, whatever) is driven by the interaction and the sequence organization, and not by an individual’s cognition.” This perspective on cognition can be applied to human-dog interaction as well.

## 5 Summary of the analysis

The first analytic section examined pre-sequences in which the dog initiated a ball game through “summons” actions, while the human responded with “answer” actions. The practices employed by the dog included “offering” the ball by dropping it on the ground. The human utilized practices such as picking up the ball or preparing the ground for the game to indicate “agreement.” The second analytic section highlighted main sequences in which the human performed “deliver the object” actions, and the dog responded with “receive the object” actions. The practices used by the human included “throwing/kicking the object,” while the dog’s practices involved “catching the object by jumping horizontally or vertically.” The third section investigated a situation in which the human disrupted the dog’s expectations regarding the next action. In this case, the “summons” action did not receive the expected “answer” actions. Consequently, the dog first attempted to repair the situation by moving to the side and then observed that this attempt did not elicit any response. The dog then deployed a second repair initiator, which ultimately succeeded, allowing the human to perform the expected repair. Additionally, the human employed the following turns of talk: the particle *no,* indicating transition in the pre-sequence, and the particle *no ni,* marking the approaching end of the main sequence. These turns demonstrate how the dog’s meaningful behavior is consequential for the human’s understanding of the ongoing activity as a “ball-throwing exercise,” with the human recognizing the dog’s agenda across the pre-sequence and into the main sequence. Furthermore, the human called the dog’s name during the play.

## 6 Discussion

Interspecies pragmatics is based on the pragmatic approach that explores language both as a form and in relation to behavior. The empirical evidence of behavior is interpretative and cognitive. Cognitive dimensions are introduced to interspecies pragmatics in this study; these dimensions are integral to the empirical evidence of meaningful behavior. At the beginning of the article, the question was posed: What kind of evidence could illustrate the involvement of animal actions in human meaning-making during interaction?

We identified two sequential occurrences in pre-sequences where human and non-human animal meaning-making processes intertwine: (1) the moment after the human’s expected response in the second position arrives, allowing the dog to proceed to the third position closing turn (dog runs) or directly to the main sequence (dog remains in place), and (2) the moment when the dog initiates a repair due to the lack of an expected answer by the human. What these occurrences have in common is that they emphasize the dog’s expectations regarding the human’s action in the second position of the pre-sequence. From the main sequence, we can discern what the pre-sequences were about: they invited the human to play ball.

In the first case, Extracts (1–3) demonstrate that the dog secures the human’s attention and then acts after the human displays multimodal signs for the dog to agree with the agenda initiated by the dog. After securing the human attention, the dog understands that they are going to play and therefore proceeds to the third position closing turn or closes the pre-sequence. Following Drew (2005, p. 181), we can treat the interaction where meaning-making processes occur as “a source of cognition” for interspecies participants. In the context of interspecies pragmatics, which considers evidence of psychological dimensions, cognition is sequentially situated within the relationship between the pre-sequence and the main sequence, indicating how occurrences in the pre-sequence materialize in the main sequence. Cognition is grounded in the sequential development—from the dog’s expectations to the highly coordinated act of catching a flying ball—making the entire exercise possible through cooperation with the other participant.

The expectations of the dog in pre-sequences refer to cognitive dimensions. Schegloff (2006, p. 87) argues that pre-sequences “foreshadow” the agenda that the presenter of a “pre-” had when deploying the “pre-.” Following this line of reasoning, Levinson notes that a pre-invitation is grounded in a “sequential template [that] is a mental entity” (Levinson, 2006a, p. 51; Levinson, 2013). In this paper, we present empirical evidence that cognitive dimensions regarding the dog’s understanding that the human knows what the dog wants—specifically, for her to throw the ball (i.e., mind-reading)—and the human’s awareness of what the dog wanted, along with her understanding that the dog knew what she knew when she proceeded from the pre-sequence to the main sequence (i.e., reflexive mind-reading), are grounded in interaction. We argue that the dynamics of a pre-sequence and the related main sequence form a mental entity that enables cognitive dimensions in social interaction.

In the second case, Extracts (1–2) and (7) can be compared to determine how the dog’s expectations of the human’s next action were fulfilled in (1–2) and not fulfilled in (7). Because the expectations were not met, the dog proceeded to engage in repair activities. Saalasti et al. (2023, p. 324) argue that any semiotic resources relevant for meaning-making processes can be used in repair. From Extract (7), we observed that the human halted the pre-sequence by standing on the dog’s ball; this game move was not cooperative. The dog deployed its embodied repair initiator by moving its body to the side, then waiting, then moving to the center, and finally up toward the human’s face. After these moves, the human self-repaired by moving away from the ball, and their interaction continued. These findings contribute to the study of repair by demonstrating how other initiations of repair and self-repair are achieved in human-non-human animal interaction. Additionally, the dog’s other initiation of repair observed in the analysis suggests that repair is not exclusively a human practice. The dog used repairs to make sense of the unusual situation; in other words, it sought to restore intersubjective understanding (Schegloff, 1992). This finding contributes to the discussion of cognition and intersubjectivity in interspecies interaction (Merritt, 2021). Furthermore, in the context of interspecies pragmatics, the use of repair organization indicates that the rules of usage (Morris, 1938) depend on the dog’s understanding of the situation: where it is now and what should happen next in terms of the exercise. However, further investigation is needed to better understand repair organization as a resource for non-human animals.

As discussed in the background section, the human interaction engine pertains to human communication (Levinson, 2006b) and includes: (1) mind-reading abilities, (2) reflexive mind-reading abilities, (3) Gricean intentions in conversation, and (4) ethological grounded practices. Only Gricean intentions may not exist in human-non-human animal interaction, as such intentions are evidenced in conversation. However, human participants maintain intentionality in these interactions. Based on this study, the human interaction engine (Levinson, 2006a,b) may not be as anthropocentric as previous critiques have suggested. It may be helpful to distinguish between human interaction, human-non-human animal interaction, and non-human animal interaction. While today’s non-human animals working with humans can engage with the engine (as shown in this study), non-human animal interaction may be less prepared to do so. In any case, there are good reasons to refer to the engine simply as the “interaction engine.”

The observed data could not be captured solely using audio-based materials (see Schegloff, 2009), indicating that the definition of the “primordial site of sociality” (Schegloff, 1987) needs to be expanded. The redefined primordial site of sociality, as demonstrated in the analyzed materials, now encompasses both humans and non-human animals. The methods used to analyze the data have also been updated; video-based materials were employed to capture the embodied actions necessary for studying this redefined primordial site of sociality. Methodologically, collecting data from a first-person perspective enables us to register, transcribe, and analyze the camera movements in detail. The action camera captures video footage and moves according to the dog’s movements. This camera movement represents a new type of resource for analyzing CA materials (see Mondada, 2013). For example, in Extract (7), line 16, the phrase “the dog moves to the right-hand side” indicates that the camera has moved accordingly. Other methodological innovations include treating transcriber comments as a resource for understanding the stepwise progression of interaction and employing two transcribing conventions, thus offering a more detailed approach for focus lines and a less detailed approach for other interactions. The latter practice saves space and helps to focus on the analytically relevant lines.

While our focus was on human meaning-making processes, a few words can be said about interspecies meaning-making. Cornips and Wels (2025) argue that co-constructing meaning in human and non-human animal interactions requires knowledge, emotions, touch, and multimodality. This is achieved by meeting non-human animals “on their own terms” (Cornips and Wels, 2025, p. 3). Based on the findings of this study, it seems that, in addition to knowing what to do and when to do it—crucial for the ball-throwing exercise as an interspecies achievement—the exercise can elicit emotions such as frustration when the human behaves oddly. However, touching is not the primary focus of the exercise, as the ball mediates their multimodal engagement. From the insights gained in this study, we can state that pre-sequences and the related main sequences establish a mental template that facilitates the use of repair organization: the mental template grants access to interactional vehicles, such as repair organization, for studying non-human animals’ thinking, knowing, and emotions. These vehicles allow us to find ways to meet non-human animals on their own terms. We can expand on what Saalasti et al. (2023, p. 324) propose regarding meaning-making processes used in repair. They suggest that any semiotic resources relevant to meaning-making processes can be utilized in repair organization. From an interspecies pragmatics perspective, not only repair activities but turns in general could be seen as laminated packages (Goodwin, 2013) that serve as fleeting semiotic resources for meaning-making processes in interaction. For example, the focus lines in this study could be seen as laminated packages illustrating how several modalities of action were present only momentarily. This understanding could shift away from human speech-centered approaches to interaction (e.g., talk-in-interaction) toward a simpler turns-in-interaction approach, which would be more inclusive of non-human animal participants.

Previous research on dog creativity has found that an unpredictable ball and uncooperative game moves can elicit opportunities for novel activities (e.g., Mitchell, 2015). In this study, we consider the dog’s actions during their walking sessions, which lead to pre-sequences and the dog’s orientation to repair the “non-working” pre-sequence, as creative. Behaviorist accounts suggest that the dog was conditioned to drop the ball when a stimulus was provided by the human (e.g., the human calling the dog’s name in Extract 2 or using a dog clicker). However, it would be difficult for behaviorist accounts to explain the dog’s behavior in Extracts (1, 3, and 7), where the dropping of the ball occurs before any stimuli. As a side note, each of these extracts demonstrates different outcomes for the dropping of the ball. Based on these observations, behaviorist accounts are likely insufficient for explaining the data.

Finally, some remarks are warranted regarding the strengths and weaknesses of the research setting that supports these bold claims. The strengths include empirical data that were rigorously analyzed using CA methods. The findings may be generalized, suggesting that the animal actions reported in this study could be observable in any dog owner’s interactions with their dog during ball games. However, the weaknesses stem from the limited number of participants involving in this study.

## 7 Conclusion

This study examined animal actions and their involvement in human meaning-making processes, utilizing empirical materials from ball-throwing sessions with dogs to demonstrate how non-human animal participants initiate pre-sequences, engage in main sequences with humans, and attempt to repair misunderstandings. Psychological dimensions relevant to the evidence of pragmatic investigation were considered, revealing that cognition is a feature of the pre-sequence and the related main sequence for dogs interacting with humans. These findings contribute to the rapidly growing field of interspecies pragmatics and offer implications for current debates on animal cognition and welfare. Future research on interspecies pragmatics could explore not only humans and dogs but also other non-human animal species, encompassing settings that range from dog-walking sessions to various other interactions. Investigating CA organizations present in interspecies interactions can be a fruitful avenue for future research.

## Data Availability

The original contributions presented in the study are included in the article/Supplementary material, further inquiries can be directed to the corresponding author.

## Footnotes

1 With human and dog projects described by Mitchell (2021: Table 1), the analyzed ball games in this paper begin with (1) the dog project: “Retrieve object” (i.e., the dog makes its ball available to the human). After the human has received the ball, the dog project (2) “Runaway” may occur, where the dog runs away from the human. (3) Human’s project: “Throw object” involves delivering the ball back to the dog. (4) Dog’s project: “Chase object” ends the ball game; the dog obtains the moving ball.
