# Supplementary material for: Animal actions and their involvement in human meaning-making processes in interaction
Source: Front Sociol. 2026 Jun 12;11:1816762. doi: 10.3389/fsoc.2026.1816762 (PMC13303903; doi:10.3389/fsoc.2026.1816762)
Supplement: Supplementary file 1 [file Data_Sheet_1.pdf]

Supplementary Material

Transcription conventions

Jefferson's conventions (2004)

(2.3)	Time in seconds
((text))	Transcriber's comments
FIG	figure

Simonen's conventions (2023)

+	Divides turns of talk and embodied actions into analytic segments (time in milliseconds)
–	Indicates actions that continue across analytic segments
#	Shows the position of a figure relative to the duration of the segment
->	Refers to actions that continue across transcript lines

Abbreviations used in both conventions

BAL:	Ball (non-human participant)
DOG:	Dog (embodied actions)
HUM:	Human (embodied actions)
hum:	Human (speech)
